# *In Vitro* Antileishmanial, Trypanocidal, and Mammalian Cell Activities of Diverse *N*,*N′* -Dihetaryl Substituted Diamines and Related Compounds

**DOI:** 10.3797/scipharm.1205-14

**Published:** 2012-10-14

**Authors:** Sandra M. Leal, Diego F. Amado, Vladimir V. Kouznetsov, Patricia Escobar

**Affiliations:** 1 Centro de Investigación de Enfermedades Tropicales, CINTROP, Departamento de Ciencias Básicas, Escuela de Medicina, Universidad Industrial de Santander, Bucaramanga, Colombia.; 2 Laboratorio de Química Orgánica y Biomolecular, Escuela de Química, Universidad Industrial de Santander, Bucaramanga, Colombia.

**Keywords:** *Leishmania (Leishmania) infantum*, *Leishmania (Viannia) panamensis*, *Trypanosoma cruzi*, Mammalian cell toxicity, *N,N′*-Dihetaryl-alkyldiamine derivatives, Drug discovery

## Abstract

The leishmaniasis and Chagas diseases constitute a serious public health problem worldwide with few and ineffective treatment options. The search for new antiparasitic candidates at the initial steps of drug discovery and development is still necessary. The synthesis of 22 *de novo* synthetized *N*,*N′*-dihetaryl-alkyldiamine derivatives and *in vitro* antiparasitic activity were evaluated for the first time against intracellular and extracellular forms of *Leishmania (Leishmania) infantum, L. (Viannia) panamensis, L. (Leishmania) amazonensis,* and *Trypanosoma cruzi*. Additionally, the toxicity on mammalian cells was determined. Some of these substituted *N*,*N′*-diamines (25–35 % of the tested compounds) showed interesting results against free-living forms of parasites with activities at the inhibitory concentration (IC*_50_*) level of 1.96 to 28.83 μM for *L. (L.) infantum* promastigotes and IC_50_ of 0.02 to 5.31 μM for *T. cruzi* epimastigotes. No activity at the IC_50_ level on intracellular amastigotes of *T. cruzi* was observed. However, *N*^1^,*N*^2^-dibenzylethane-1,2-diamine **5a** revealed an important activity against the intracellular amastigotes of *L. infantum* (IC_50_ 25.42 μM ±0.33) and *L. panamensis* (IC_50_ 58.20 μM ±3.23)*,* while their analogue *N^1^,N^4^*-dibenzylbutane-1,4-diamine **5c** resulted in activity only against *L. panamensis* (IC_50_ 11.19 μM ±0.20) without toxicity on Vero and THP-1 mammalian cells. The active compounds against intracellular parasites with low toxicity in mammalian cells may be considered for future studies in experimental models.

## Introduction

The leishmaniasis and Chagas diseases represent a major public health problem in various tropical and sub-tropical regions. They are produced by digenetic protozoa living as extracellular parasites on their respective insect vectors and as intracellular amastigotes inside mammalian host cells. Leishmaniasis is caused by many species of protozoa belonging to the genus *Leishmania* transmitted by the bite of the phlebotomine sandfly. Leishmaniasis currently threatens 350 million people in 88 countries around the world, where 12 million people are affected and 2 million new cases occur each year [1]. There are no effective vaccines and current chemotherapy is based on the use of pentavalent antimonies followed by amphotericin B (AmB), pentamidine isothionate, paramomycin, and miltefosine. They are still not the ideal antileishmanial drug and problems such as low efficacy, high toxicity, and cost and/or high risk of induced resistance are reported [2].

Chagas disease, also known as American Trypanosomiasis, is one of the most serious protozoan diseases that occurs throughout Latin America. It is produced by *Trypanosoma cruzi,* a flagellate protozoan which is transmitted to humans and other mammals mostly by hematophagous insects of the Reduviidae family, but also congenitally, orally, or by blood transfusion [3]. There are 90–100 million people at risk, with 16–18 million cases [4]. There are no prophylactic drugs to prevent infection, and current chemotherapy is based on two drugs (nifurtimox and benznidazole), effective only for recent infections and for the short-term chronic phase of the disease. Both drugs have severe side-effects, (allergic dermopathy, anorexia, vomiting, peripheral polyneuropathy, and psychic alterations) requiring long courses of treatment, and exhibit variable efficacy [5]. Gentian violet has been used for the prevention of Chagas disease by blood transfusion and some drugs originally developed to treat fungal infections (itraconazole, ketoconazole, posaconazole, and ravuconazole) were evaluated in clinical trials [5, 6].

Thus, the increasing problems derived from the employment of the currently used drugs in treating the leishmaniasis and Chagas diseases have resulted in an urgent need for novel, non-toxic, selective, and cost-effective new drug candidates in this area. During the last few years, a new incentive to discover antileishmanial and antitripanosomal drugs has arisen. Several aspects such as advances in the knowledge of the biology and genome of parasites, bioinformatics and chemical techniques, networks, partnerships, and consortia have supported the development of new antileishmanial agents. Currently, the development of both synthetic and natural drugs have relevant importance in the search of new therapeutic alternatives [6, 7]. In recent years, traditional medicine has great importance in the field of chemotherapy against tropical diseases as an alternative to treatment. Essential oils, plant extracts, oils components, among others with antiparasitic activity, have been tested against trypanosomatid, however, few natural compounds continue to be tested in *in vivo* studies [8, 9].

The synthesis of a wide number of polyamine analogs has been prompted and some promising compounds with trypanocidal properties have been obtained. Polyamines are a group of organic cationic molecules practically present in all living organisms. They play vital roles in cell proliferation and differentiation and macromolecular biosynthesis [10]. In trypanosomatids, the mechanism of polyamine transport and synthesis has been studied and important differences between mammalian and parasitic polyamine metabolism have been established, supporting the use of polyamine biosynthesis inhibitors or polyamine derivatives as a promising strategy in the search for antiparasitic drugs [10–13]. A series of *N*,*N*′-bis(benzyl) substituted polyamine analogs were found to inhibit *L. donovani*, *T. cruzi,* and *Plasmodium falciparum* parasites in *in vitro* and animal experimental models [14–16]. Recently, a series of novel diamine derivatives have demonstrated activities against both *L. donovani* promastigotes and *P. falciparum*[17], while diverse diamines containing a fatty chain attached to the ethylenediamine fragments or 1,2-cyclohexanediamine derivatives were active on *L. amazonensis*, *L. braziliensis,* and *L. chagasi* promastigotes [18, 19]. In addition, *N*-alkyl lipophilic diamines were highly active against *L. amazonensis* and *L. chagasi* promastigotes, where the ethylenediamine derivative contains a 12-carbon alkyl chain substitute [20]. Polyamine derivatives such as *bis*-naphthalimidopropyl putrescine, spermidine, and spermine were active on *L. infantum* promastigotes [21].

With these precedents in mind and as a part of a screening program for new molecules with antiparasitic activities [22, 23], the aim of this project was to evaluate the *in vitro* biological activity against parasites and mammalian cells of several *N*,*N′*-dihetaryl substituted diamines and related compounds derived from ethylenediamine, 1,3-propane-diamine and putrescine.

## Results and Discussion

In an attempt to find different small heterocyclic molecules active against protozoan parasites, we decided to prepare some *N*,*N′*-dihetaryl substituted diamines *de novo* whose antiparasitic properties are unknown at the moment. Thus, using a common synthetic method that includes the formation of the *N*,*N′*-dihetarylidenalkane-diamines **4a–k** from the primary diamines (ethylenediamine **1**, 1,3-propanediamine **2**, putrescine **3**) and benz-aldehydes (pyridincarboxyaldehydes) as precursors, and the imine’s reduction by treatment with excess NaBH_4_ in methanol, the desired *N*,*N’*-alkane-diamines with aryl (comp. **5a–e**) or pyridinyl (comp. **5h–j**) moities were easily synthetized [24–27]. The *bis-*thiazolidinones **6a,c** and **6f–k,** joined through ethylene spacers, were obtained via the condensation of α-mercaptoacetic acid and diimines **4** following our previously reported protocol [28]. The simple diamine precursors **1–3** and the *N*^1^,*N*^4^-dibenzylbutane-1,4-diamine **5c** were chloroacethylated with chloroacethyl chloride at −40 °C to achieve the respective chloroacethyl amides **7a–c** and **8** (Sch. 1).

Having these diamine molecules on our hands, we proceeded to perform biological assays. First, *in vitro* activity on *L. (L.) infantum* promastigotes and *T. cruzi* epimastigotes of the selected molecules **5–8** was studied. Analyzing the obtained results (Tab. 1), we found that four compounds, **5a–c** and **8**, were active on promastigotes of *L. chagasi* with activities ranging from IC_50_ 1.96 ± 0.07 to 12.19 ± 2.45 μM (p<0.05). Four compounds, **5a,d,e** and **8**, exhibited potent activity (from IC_50_ 0.02 ± 0.004 to 2.54 ± 0.08 μM) against *T. cruzi* epimastigotes, being more active than the reference drug (nifurtimox, p<0.05). Moreover, their selective index (SI) was higher than 6.4 indicating parasite-selective activity. The benzyl disubstituted diamine **5a**, having two carbons as a spacer, and the dichloroacetamide dibenzyl substituted diamine **8**, with four carbons as a spacer, were active against both parasitic free-living forms. In contrast, dibenzyl diamine analogues **5b,d** were more selective towards the epimastigotes of *T. cruzi.* Surprisingly, a series of new *bis-*thiazolidinones joined through a methylenic spacer (comp. **6a,c** and **6f–k**) appeared to be inactive. Other authors have reported similar results in terms of the antiparasitic activity of N, N-substituted diamines. Caminos et al, [29] reported an IC_50 of_ less than 10 μM for these compounds against free-living forms of *L. donovani* and *T. cruzi* and the bloodstream form of *T. brucei* with low toxicity on Vero cells. The activities against intracellular parasites were not evaluated in this study. Diverse diamines have been actives also on *L. amazonensis*, *L. braziliensis,* and *L. chagasi* promastigotes with IC_50_ values <10 μg/mL [18, 19].

Following the results from the mammalian cell toxicity tests on Vero and THP-1 cells, we could note that compound **8** was partially toxic to THP-1 cells (CC_50_ 25.05 ± 0.35 and CC_90_ > 207.60), and dichloroacetamides **7a,c** were toxic on Vero cells (CC_50_ 25.69 ± 1.22 and 37.11 ± 0.45 μM and CC_90_ 49.50 ± 1.87 and 77.57 ± 3.15 μM, respectively) as shown in Table 1. In general, all compounds tested showed low toxicity, however, other studies of toxicity such as genotoxicity, teratogenicity, mutagenicity, among others, are necessary to continue with the following experimental phases *in vivo*. Furthermore, compounds active in both free and intracellular forms showed an IS> 6, indicating that biological activity could be directed primarily against the parasite and not against host cells suggesting the selectivity of the compound.

The second part of our study consisted of bio-screening on *in vitro* activity on intracellular amastigotes of both *Leishmania* spp and *T. cruzi* parasites. From the overall activity of tested molecules, only *N*,*N′*-hetaryl diamines **5a** and **5c** were active on the intracellular live forms of *Leishmania* parasites. Any compound was active on the intracellular form of *T. cruzi*. The *N,N′-*dibenzylethane-1,2-diamine **5a**, having a calculated log *P* = 3.58, showed the best antiparasitic activity on the intracellular amastigotes of *L. (L.) infantum* infecting THP-1 macrophages (IC_50_ values of 25.42 ± 0.33 μM, SI = 9.39, p<0.05) compared with the other species of *Leishmania*. It was also partially active on *L. panamensis*. Interestingly, its closer pyridinylalkane-diamine analogues (comp. **5h–j**) did not possess these properties. In contrast, the dibenzyl diamine derivative **5c** (log *P* = 3.92) with four carbons as a spacer showed activity on the intracellular amastigote of *L. panamensis* (Tab. 2). These active compounds may be assessed in future studies in animal models since polyamine analogs have shown in previous reports to inhibit *L. donovanni* in experimental models [14].

## Conclusion

This work described the antiparasitic and cytotoxic evaluation of several lipophilic *N*,*N′*-dihetaryl substituted diamines prepared from simple diamines by the iminonization reaction and reduction reaction. During our biological study, we identified several *N*,*N′*-diamines with excellent *in vitro* results against the *Leishmania spp and/or T. cruzi* parasites. The activity data suggest that the methylenic spacer bearing two to four carbons may be effective on antiparasitic diamines while the ethane-1,4-diamine skeleton is disubstituted with dibenzyl (comp. **5a**) fragments. Also, the dichloroacetamide dibenzyl substituted with four carbons as a methylenic spacer (comp. **8**) did work well with better results than the other diamine derivatives.

Correlation between the calculated log *P* values and antiparasitic activities data showed that log *P* parameters were, in general, similar for active as well as for inactive compounds. However, it could be noted that except the compound **5d**, which has a log *P* less than 2 (1.52) and compound **5e** with log *P* 1.96, molecules **5a–c** and **8** with the highest potencies against *T. cruzi* and/or *L. chagasi* had a partition coefficient between the range of 3.58 and 3.92. From these data, we could deduce that the lipophilicity of the tested disubstituted diamines would not have a direct influence on their antiparasitic activity. These results are important data for the design and synthesis of new related diamine derivatives, which could become leaders in the development of antiparasitic agents.

## Experimental

### Chemicals

Solvents and common reagents were obtained from Merck and Aldrich and were used without further purification. The reaction progress was monitored using thin layer chromatography on a silufol UV254 TLC aluminum sheet. The chemical purity of obtained compounds **5–8** was confirmed using elemental analyses, performed on a Perkin Elmer 2400 Series II analyzer, that were within ± 0.4 of theoretical values. Their spectral and physical properties were in agreement with those reported in literature [22–26].

### Biological reagents

Amphotericin B, phorbol mirystate acetate (PMA), dimethylthiazoldiphenyltetrazolium bromide (MTT), HEPES, adenosine, and hemin were purchased from Sigma-Aldrich, USA. Nifurtimox was donated kindly by Professor Simon Croft at the LSHTM, London, UK. Dimethylsulphoxide (DMSO) was obtained from Carlo Erba (Reagenti Rodano, Italy). Culture medium RPMI 1640, fetal calf serum (FCS), and trypsin-EDTA were obtained from Gibco (Grand Island, NY, USA). Stock solutions of the compound and the reference drugs Amphotericin B and nifurtimox were prepared on DMSO (final concentration 0.1%, v/v). Work solutions were made in culture medium immediately before the assays.

### Parasite and mammalian cells

Promastigotes forms of *L. chagasi* (MHOM/BR/74/PP75) syn, *L. (L: infantum), L. panamensis* (MHOM/PA/71/LS94), and *L. amazonensis* (MHOM/BR/73/LV78) parasites were grown in RPMI 1640 medium supplemented with 10% heat-inactivated fetal cow serum (hiFCS) and 0.5 mg/L hemin at 28 °C. Epimastigotes forms of *T. cruzi* (320I01 strain, kindly donated by K.P. Luna, CINTROP-UIS) [30] were maintained on Liver Infusion Tryptose (LIT) medium with 10% hiFCS at 28 °C.

Vero cells (CCL-81, ATCC) and THP-1 (TIB-202, ATCC) mammalian cells were cultivated in RPMI 1640 medium supplemented with 10% of hiFCS at 37 °C, 5% CO_2_-95% air mixture.

### Parasites assays

Epimastigotes of *T. cruzi* and promastigotes forms of *L. (L.) infantum* (5 × 10^5^ parasites/mL) were placed in flat-bottomed 96-well microtiter plates (Becton Dickinson, New Jersey, USA) and treated with a three-fold dilution series of the compound (from 0–350 μM) and reference drugs (1.2–100 μM) for 72 h at 28 °C. The inhibition of parasite growth was microscopically determined by counting viable (live) parasites in a haemocytometer using yellow eosin as vital dye; live parasites (uncolored) and dead (orange) were counted. All of the experiments were performed with parasites in their logarithmic phase of growth [31].

For the amastigotes assays, adherent Vero cells or THP-1 macrophages (after transformation with 10 ng/mL PMA) were infected with tissue-derived trypomastigotes or stationary phase promastigotes, respectively, at a ratio of 10:1 and maintained in a 5% CO_2_-95% air mixture at 37 °C. After 24 h, infected cultures with >50% of infected cells were incubated with the compounds (from 3–300 μM) in a three-fold dilution series for 72 h. Drug activity was determined from the % of infected cells in treated and untreated cultures in fixed methanol and Giemsa stained preparations. Parasites’ assays were performed twice and each tested concentration was tested in triplicate [31].

Amphotericin B and nifurtimox were used as reference drugs in *Leishmania* and *T. cruzi* respectively. Negative controls were used without the drug (culture medium alone) in each experiment.

### Mammalian cell assays

Vero cells and THP-1 cells transformed into adherent macrophages with PMA in 96 microwell plates where they were treated with compounds (0–350 μM) during 72 h at 37 °C, 5% CO_2_-95% air mixture. The viability of the cells was evaluated by using the tetrazolium-dye (MTT) colorimetric method. After incubation of the cells with the MTT (5mg/mL) reagent for 4 hours, DMSO was added. The samples were read using an ELISA plate reader at a wavelength of 580 nm. The amount of color produced is directly proportional to the number of viable cells. The percentage of cytotoxicity was calculated by the equation: cytotoxicity (%) = (OD control group−OD treatment group)/OD control group × 100 [31].

## Statistical analysis

The antileishmanial and antitrypanosomal activities were expressed as the concentration able to inhibit parasitic growth by 50% (IC_50_) and 90% (IC_90_) after a 3-day incubation period. The cytotoxicity of mammalian cells was expressed as the concentration able to kill cells by 50% (CC_50_) and 90% (CC_90_) after a 3-day incubation period. The data were calculated by linear regression using the software (Msxlfit; GO Business Solution, Guildford, UK). The selectivity index (SI) was calculated dividing CC_50_/IC_50_. Each experiment was repeated twice and the values represent the results of one representative experiment. Active compounds were considered with IC_50_ <25μM and IC_90_ <100 μM, partially active with IC_50_ >25 μM and IC_90_ <100 μM, and not active with IC_50_ >25 μM and IC_90_ >100 μM according to laboratory criteria. The analysis to determine the statistical differences in biological activity was performed using the analysis of variance (ANOVA) and the Bonferroni post-test. We also performed Student’s t test for comparison between the two variables, using GraphPad Prism software version 5.03. P values <0.05 were considered to be statistically significant.

Theoretical values of the partition coefficient (log *P*) were calculated in the ACD Log PDB (ACD/Labs 6.00) program. The log *P* of each compound was calculated in order to correlate the qualitative structure-activity relationships with quantitative parameters. It is known that the log *P* of a drug or drug-like substance is an indicator of the compound lipophilicity and solubility, and it is a useful parameter in drug discovery and development and is issued to predict the transport properties across cell membranes, to establish quantitative structure-activity relationships, and as an indicator of protein-binding characteristics [24].

## Figures and Tables

**Sch. 1.**
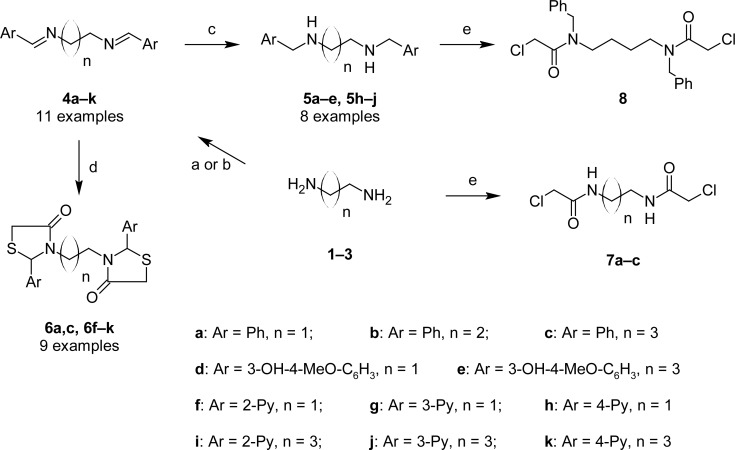
*Synthesis of functionalized diamines. Reagents and conditions*: (a) ArCHO, EtOH, rt, 1 h, 61–100%; (b) ArCHO, EtOH, reflux, 7 h, 64–100%; (c) NaBH_4_, MeOH, rt, 3-5 h, 53–93%; (d) HSCH_2_COOH, MeCN, 5 °C, 1 h, 33–68%; (e) ClCH_2_COCl, CH_2_Cl_2_, Et_3_N, 40 °C, 2 h, 61–86%

**Tab. 1.** *In vitro* activity of *N*,*N′*-dihetaryl substituted diamines **5–8** against *Trypanosoma cruzi, Leishmania (L.) infantum,* and mammalian cell lines.

**Cpd.**	**Structure/Molecular Formula**	**Log *P***	**μM**					
			***L. (L) infantum***		***T. cruzi***		**Mammalian cells**	
			**promastigotes**		**epimastigotes**		**THP-1**	**Vero**
			**IC_50_ ± SD^a^**	**SI^b^**	**IC_50_ ± SD^a^**	**SI**	**CC_50_ ± SD^c^**	**CC_50_ ± SD**
**5a**	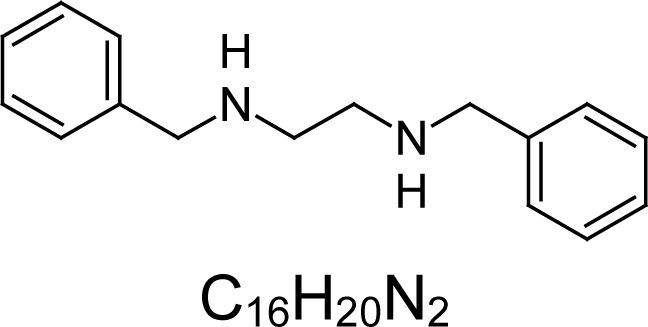	3.58	12.19 ± 2.45	19.58	2.54 ± 0.08	29.62	238.74 ± 17.93	75.26 ± 2.57
**5b**	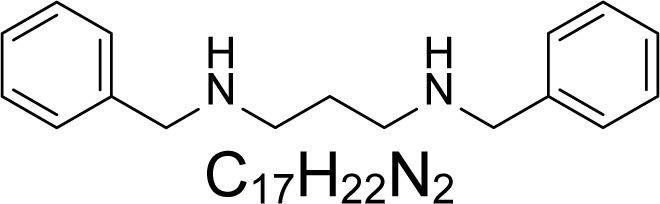	3.62	1.96 ± 0.07	86.29	5.35 ± 0.27	64.2	169.13 ± 29.76	343.48 ± 29.37
**5c**	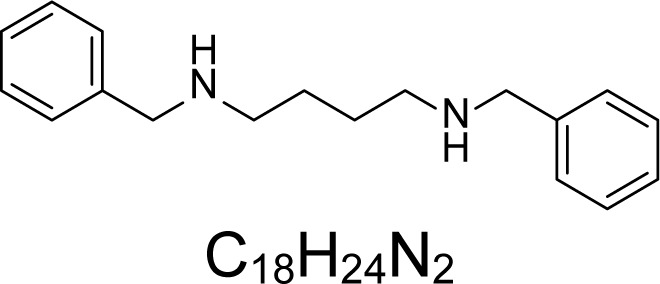	3.92	11.23 ± 0.11	12.75	112.00 ± 1.66	0.53	143.28 ± 13.35	60.37 ± 2.42
**5d**	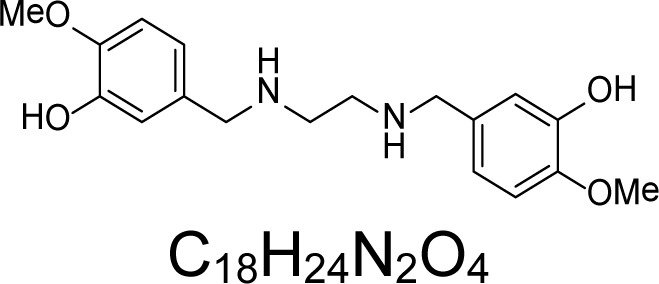	1.52	40.24 ± 5.21	>7.48	0.88 ± 0.06	>300	>300	>300
**5e**	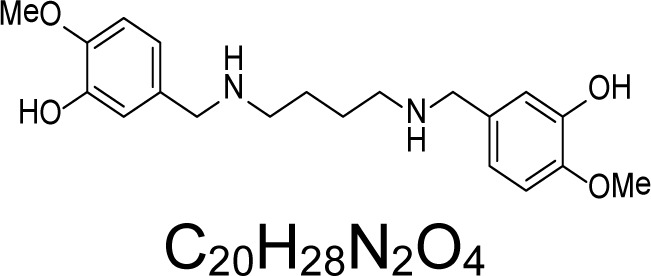	1.96	>250	>0.89	1.22 ±0.08	>200	247.91	>250
**5h**	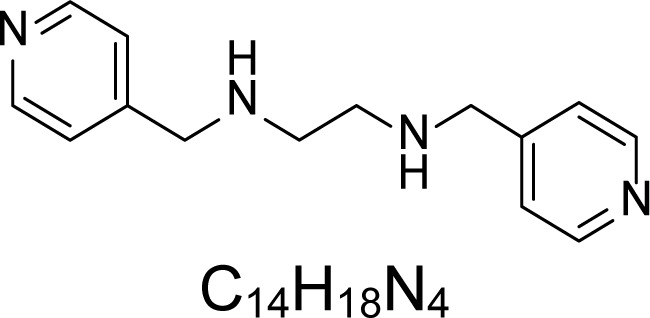	0.58	180.54 ± 1.89	>2.28	>250	>0.86	>412.67	355.64 ± 37.05
**5i**	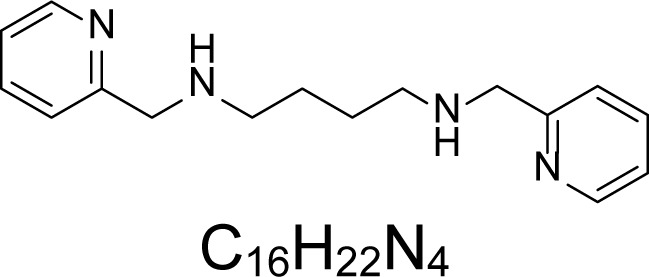	0.93	28.83 ± 5.14	>12.82	>250	>0.35	>300	131.52 ± 12.31
**5j**	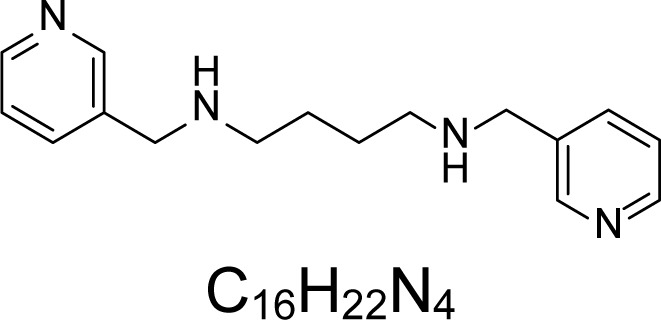	0.93	44.19 ± 10.54	>8.36	>250	>1	>300	>300
**6a**	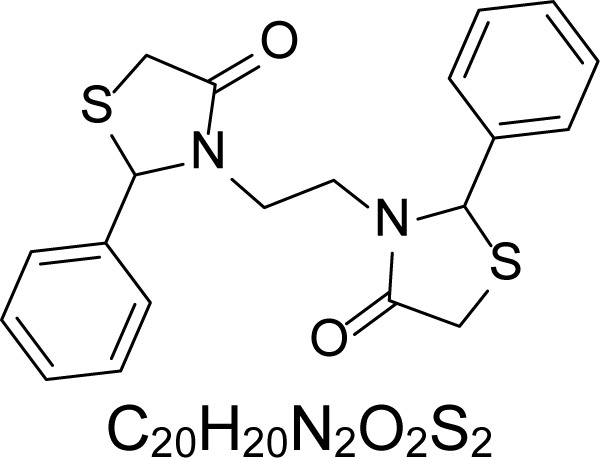	4.46	135.8 ± 6.96	>1.91	237.21 ± 12.14	0.72	>250	170.84 ± 3.77
**6c**	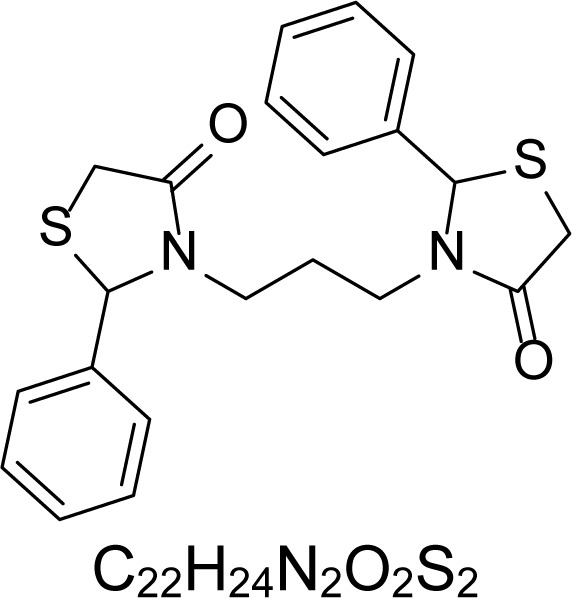	4.57	>250	>1.0	>250	>1.01	>250	>250
**6f**	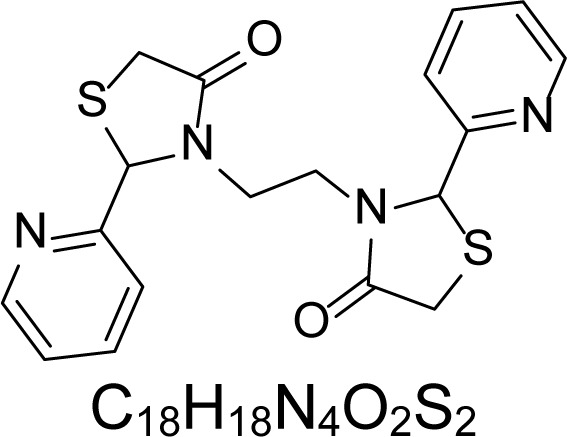	1.48	176.42 ± 16.04	>1.46	161.22 ± 5.58	>1.60	>250	>250
**6g**	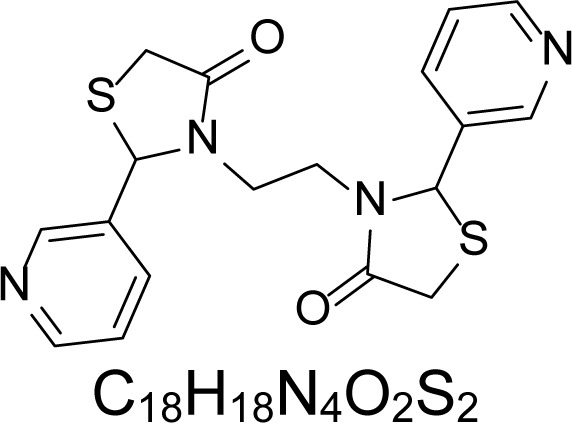	1.48	>250	>1.0	>250	>1.0	>250	>250
**6h**	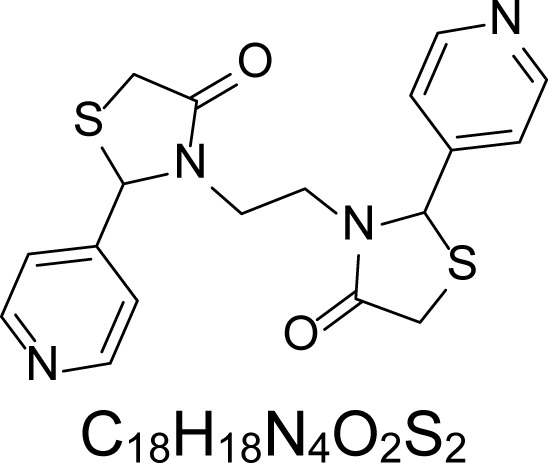	1.47	>250	>1.0	>250	>1.0	>250	>250
**6i**	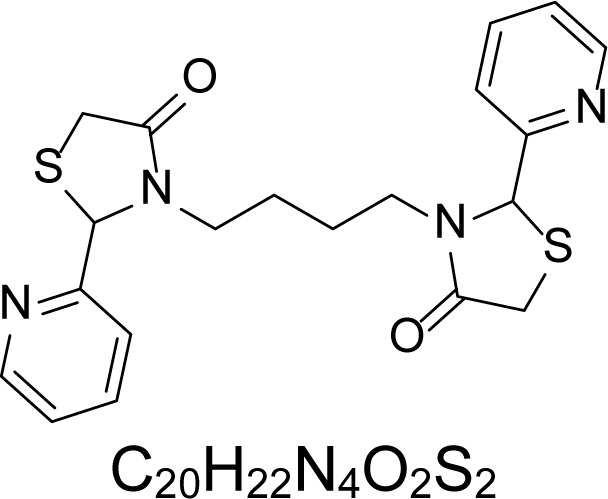	1.59	>250	>1.0	>250	>1.0	>250	>250
**6j**	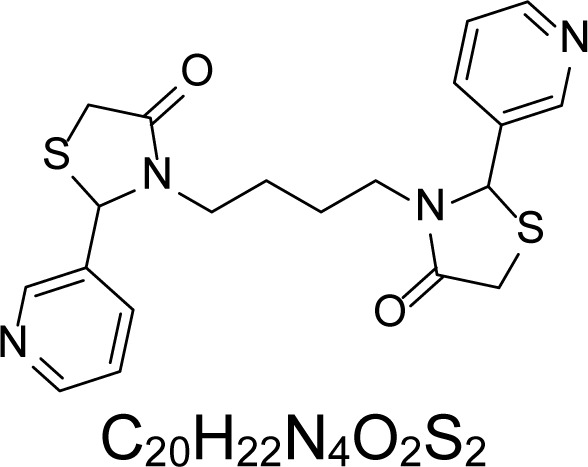	1.59	>250	>1.0	235.03 ± 3.06	0.32	>250	75.36 ± 1.80
**6k**	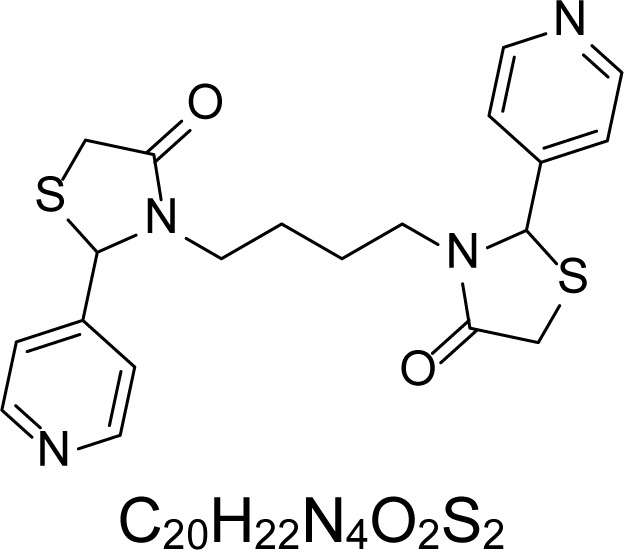	1.58	>250	>1.0	>250	>1.0	>250	>250
**7a**	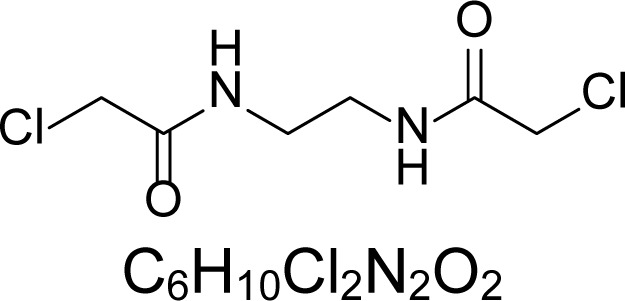	−1.04	176.02 ± 18.97	>2.66	65.94 ± 5.54	0.38	>300	25.69 ± 1.22
**7b**	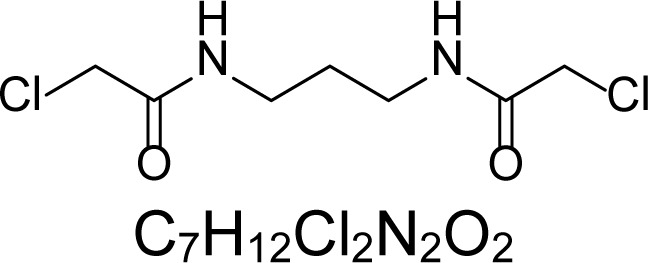	0.08	76.43 ± 1.05	2.07	1.01 ± 0.13	32.35	158.37 ± 4.53	32.68 ± 6.56
**7c**	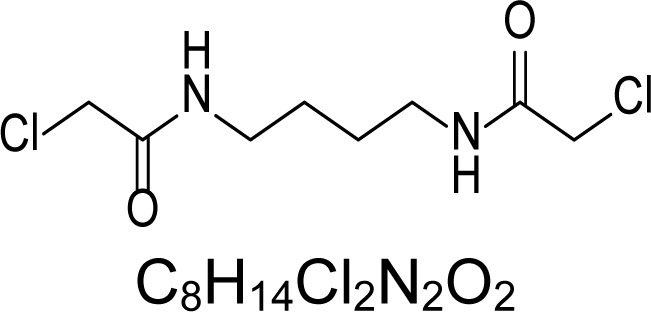	−0.45	132.17 ± 7.63	2.76	54.21 ± 1.16	0.68	365.67	37.1 ± 0.45
**8**	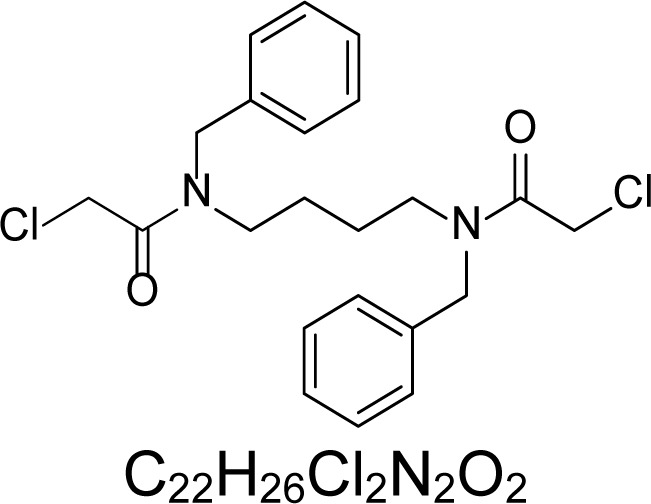	3.68	3.91 ± 0.01	6.4	0.02 ± 0.004	>11.87	25.05 ± 0.35	>250
**AmB**	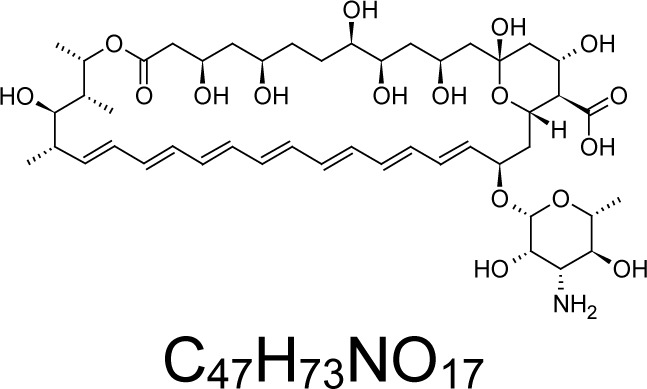	−2.489	0.014 ± 0.001	948.57	ND^d^	ND	13.28 ± 1.72	ND
**Nif**	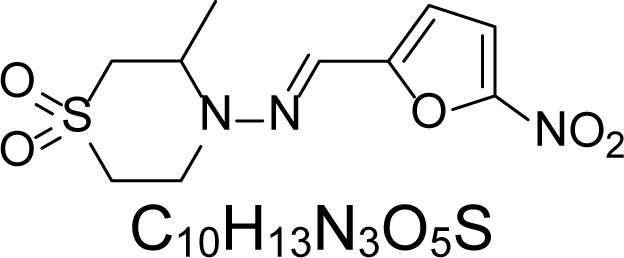	0.709	ND	ND	2.71 ± 0.05	28.23	ND	76.53 ± 3.20

a Inhibitory Concentration 50 (concentration inhibiting 50% of the parasites);

b Cytotoxic Concentration;

c Selective Index;

d Not determined.

**Tab. 2.** *In vitro* activity of *N*,*N′*-dihetaryl substituted diamines against intracellular amastigotes of *Leishmania* spp and *Trypanosoma cruzi.*

**Cpd.**	**μM ± SD^a^**					

	***L. (L.) infantum* (48.58%)^b^**			***L.(V.) panamensis* (78.3%)^b^**		
	**IC_50_^c^**	**IC_90_**	**SI^d^**	**IC_50_**	**IC_90_**	**SI**

**5a**	25.42 ± 0.33	59.54 ± 2.78	9	58.20 ± 3.23	88.13 ± 1.85	5
**5c**	T^e^			11.19 ± 0.20	41.68 ± 0.76	4
**AmB**	0.045 ± 0.007	0.138 ± 0.023	360	0.032 ± 0.002	0.20 ± 0.002	420

**Cpd.**	**μM ± SD^a^**					

	***L. (L.) amazonensis* (58.6%)^b^**			***T. cruzi* (98.1%)^b^**		

	**IC_50_**	**IC_90_**	**SI**	**IC_50_**	**IC_90_**	**SI**

**5a**	49.27 ± 4.70	130.8 ± 9.14	6	131.20 ± 0.92	>123	0.48
**5c**	ND^f^	ND		96.05 ± 13.48	106.25 ± 13.07	0.82
**AmB**	0.082 ± 0.002	0.20 ± 0.002	616			
**Nif**				2.34 ± 0.83	10.24 ± 1.60	55

AmB: Amphotericin B; Nif: Nifurtimox;

a Standard deviation;

b Percent of cells infected on the parasite before of treatment with compounds;

c Inhibitory Concentration 50 (concentration inhibiting 50% of the parasites);

d Selective Index;

e Toxic;

f Not determined; Each experiment was repeated twice and the values represent the results of one representative experiment (n = 3).
